# Can Following Paleolithic and Mediterranean Diets Reduce the Risk of Stress, Anxiety, and Depression: A Cross-Sectional Study on Iranian Women

**DOI:** 10.1155/2023/2226104

**Published:** 2023-03-03

**Authors:** Behzad Zamani, Mobina Zeinalabedini, Ensieh Nasli Esfahani, Leila Azadbakht

**Affiliations:** ^1^Department of Community Nutrition, School of Nutritional Sciences and Dietetics, Tehran University of Medical Sciences, Tehran, Iran; ^2^Endocrinology and Metabolism Research Center, Endocrinology and Metabolism Research Institute, Tehran University of Medical Sciences, Tehran, Iran; ^3^Students' Scientific Research Center (SSRC), Tehran University of Medical Sciences (TUMS), Tehran, Iran; ^4^Diabetes Research Center, Endocrinology and Metabolism Clinical Sciences Institute, Tehran University of Medical Sciences, Tehran, Iran; ^5^Department of Community Nutrition, School of Nutrition and Food Science, Isfahan University of Medical Science, Isfahan, Iran

## Abstract

**Background:**

Psychiatric disorders have been a challenge for public health and will bring economic problems to individuals and healthcare systems in the future. One of the important factors that could affect these disorders is diet.

**Objective:**

In the current study with a cross-sectional design, we investigated the association of Paleolithic and Mediterranean diets with psychological disorders in a sample of adult women.

**Methods:**

Participants were 435 adult women between 20 and 50 years old that refer to healthcare centers in the south of Tehran, Iran. The diet scores were created by the response to a valid and reliable semiquantitative food frequency questionnaire (FFQ), and the psychological profile was determined by response to the Depression, Anxiety, and Stress Scale (DASS-21). The multivariable-adjusted logistic regression was applied to compute the odds ratio (OR) and 95% confidence interval (CI).

**Results:**

After adjusted for potential confounders, it is evident that participants in the highest Paleolithic diet tertile had lower odds of depression (OR = 0.21; 95% CI: 0.12, 0.37: *P* < 0.001), anxiety (OR = 0.27; 95% CI: 0.16, 0.45: *P* < 0.001), and stress (OR = 0.19; 95% CI: 0.11, 0.32; *P* < 0.001) in comparison to the lowest tertile. Furthermore, those in the third tertile of the Mediterranean diet score were at lower risk of depression (OR = 0.20; 95% CI: 0.11, 0.36; *P* < 0.001), anxiety (OR = 0.22; 95% CI: 0.13, 0.38; *P* < 0.001), and stress (OR = 0.23; 95% CI: 0.13, 0.39; *P* < 0.001) compared with those in the first tertile.

**Conclusion:**

The result of the current study suggests that greater adherence to Paleolithic and Mediterranean dietary patterns may be related with a decreased risk of psychological disorders such as depression, anxiety, and stress.

## 1. Introduction

Psychological disorders were considered one of the leading causes of disability with severe consequences [1]. Some common psychological disorders are depression, anxiety, and stress [2]. Over 300 million individuals, or 4.4% of the world's population, are depressed [3]. Globally, 7.3% of people experience anxiety, and 17.6% experience psychological discomfort [4, 5]. Women have twice as many mental problems as males [6]. Psychological disorders make people more susceptible to economic, social, and health problems [5, 7]. Psychological disorders are linked to genes and lifestyle factors, including inactivity and smoking [8].

One of the lifestyle factors that is associated with psychological disorders is nutrition [9]. The association between diet and psychological disorders has been highlighted in several studies [10]. High consumption of fruits, vegetables, nuts, legumes, and whole grains was related to a lower risk of psychological disorders [11]. Contrarily, diets heavy in red meat, processed meat, high-fat dairy items, refined grain, and high-sugar beverages are linked to increased psychological disorders [12]. Mediterranean diet (MD) as a healthy dietary pattern has a positive impact on CVD, diabetes, and neurological disorders [13].

There is no consensus on the relationship between the MD and mental health. In cross-sectional research conducted in Spain [14], adherence to the Mediterranean diet was related to a decreased incidence of depression and anxiety. In contrast, such a substantial connection was not detected [15] in a study of 1183 Australian adults. However, a significant component of this Australian sample's diet consisted of items not typically eaten in a Mediterranean-style diet. Research on female adolescents in Iran revealed a correlation between MDP adherence and decreased depressive symptoms [16]. However, a comprehensive review and meta-analysis found no significant association between the MD and depression risk in cohort studies [17]. Although a cross-sectional study in Iran identified evidence demonstrating a negative link between the MD and the risk of psychiatric disorder in both sex groups [18], no study specifically focuses on the female population. Moreover, the Paleolithic diet, which is high in fruits, vegetables, nuts, and roots and low in fried foods, grains, dairy products, salt, refined fats, and sugar, has positively affected the glycemic index and CVD risk markers [19, 20]. However, no study has so far examined the potential association of the PD with psychological disorders.

To the best of our knowledge, no study is available linking both dietary pattern PD and MD on psychiatric disorders in Iranian women. It should also be noted that the prevalence of psychological disorders in this population differs from other communities. In addition, previous research in Iran revealed that the MD adhered to nine components of mental disorders; however, the current study assessed ten components.

Given these points and the controversial findings of the MD on mental disorders, we conducted the present study to examine the association of the PD and MD with psychological disorders in a sample of Iranian women.

## 2. Methods

### 2.1. Study Design and Participants

The present study's cross-sectional design was conducted on a women population. We selected participants from clients referred to 10 healthcare centers affiliated with the Tehran University of Medical Sciences (TUMS). These centers were chosen at random from a total of 29 local healthcare centers. We determined the number of our subjects at each chosen health center concerning the overall number of people using the facility. Using the following formula, *α* = 0.05, standard deviation (*σ*) = 5.2, and estimation error (*d*) = 0.5 [21], sample size was calculated as follows: *n* ≥ [*Z*_1−*α*/2_ × *σ*/*d*]^2^. According to this formula, 416 people were needed; however, given access to data and to consider any probable exclusion, 435 participants were studied. Several healthcare centers affiliated with the TUMS were chosen randomly with a multistage cluster sampling method. Before the research, participants provided informed permission in writing. Those who did not provide informed consent and did not participate in completing the questionnaires, and those who consumed less than 500 or more than 3,500 kilocalories of energy per day, were eliminated from the research. Participants had to be healthy women between 20 and 50 years old and of Iranian descent to be included. Women who were pregnant, lactating, premenopausal, with chronic diseases such as diabetes, cardiovascular diseases, liver or kidney dysfunction, cancer, or diagnosed with a psychological were not included. Individuals taking medications affecting the mental status were also excluded. Informed consent was received from all participants. The research council of the School of Nutritional Sciences and Dietetics of the TUMS confirmed the study protocol (number: 9511468004).

### 2.2. Assessment of Psychological Profile

The psychological profile was evaluated by the short form of the Depression, Anxiety, and Stress Scale (DASS-21) [22]. Since the DASS-21 is based on a dimensional concept rather than a categorical concept of mental disorders, we cannot compare variables using the groups “depressed,” “anxious,” and “stressed,” which would be appropriate for a clinical diagnostic tool.

The Beck Depression Inventory scale and the Depression subscale had *a* + 0.70 correlation, the Zung Anxiety Inventory and the Anxiety subscale had *a* + 0.67 correlation, and the Perceived Stress Inventory and the Stress subscale had *a* + 0.49 correlation. In addition, its reliability and validity in the Persian version have been approved previously [23].

To fill up the questionnaire, one must indicate the current state of a symptom during the previous week. Each of the three subscales of the DASS consists of seven questions. The final score was derived from the sum of the scores on three subscales. The responses are categorized as zero, low, medium, and high, with scores ranging from 0 to 3. Since the DASS-21 is the abbreviated version of the original scale (42 questions), the final score for each of these subscales should be multiplied by two [24]. For depression, anxiety, and stress, the individuals are classified into five categories based on their overall score: normal, mild, moderate, severe, and extremely severe. To categorize participants into two groups, the median score was used as the cutoff point, and they were divided into groups higher and lower than the median.

### 2.3. Assessment of Dietary Intake

A valid and reliable 168-items semiquantitative food frequency questionnaire (FFQ) was used to assess usual dietary intake [25]. An expert nutritionist collected nutritional data via a face-to-face interview. Household measures were used to convert portion sizes to gram intake. Then, macro- and micro-nutrient intakes were computed using Nutritionist IV software (First Databank Division, the Hearst Corporation, San Bruno, CA, USA, modified for Iranian foods).

### 2.4. Paleolithic Diet and Mediterranean Diet Scores Measurement

The work by Whalen et al. [26] was used to compute the PD and MD compliance scores. The food items gathered from each participant's FFQ were categorized into 14 food categories (such as vegetables, fruits, fruit and vegetable diversity score, lean meat, fish, nuts, and calcium as more PD characteristics and red and processed meat, dairy foods, sugar-sweetened beverages, baked goods, grains and starches, sodium, and alcohol as less characteristic PD). The range of PD scores was from 13 to 65, with higher values indicating more adherence to the PD.

MD scores have 11 components. As shown in Table 1, this approach was adjusted regarding dairy items, cereals, starches, and alcohol consumption for the MD score. The components of the score were transformed into quintiles of consumption, and a score of 1–5 was applied to each component. The score of each component was then summed to create the final diet pattern score. The final scores could range from 10–50 for the ten components of the MD score.

Because all research participants were Muslims and did not use alcohol, alcohol consumption was not considered a factor in these results.

### 2.5. Assessment of Other Variables

Baseline information was obtained and documented, including age, marital status, socioeconomic status (home and welfare status), education status, and use of supplements or medications using a sociodemographic questionnaire.

### 2.6. Socioeconomic Status Demographic

The socioeconomic status demographic questionnaire was used for this purpose, which included questions on marital status, education, occupation, family size, means of support, and mode of transportation. The codes were appended to each questionnaire item to generate the socioeconomic status score. The score was split into three groups, and individuals were classified according to their socioeconomic status: low, middle-class, or high [27].

### 2.7. Anthropometric Indices

The subject's body weight was assessed by a digital scale (SECA, Hamburg, Germany) which is closest to 0.1 kg while wearing light clothing and without footwear. A wall-mounted stadiometer measured standing height to the nearest 0.5 cm. The following formula was used to calculate the body mass index (BMI): BMI = weight/height^2^ (kg/m^2^).

### 2.8. Physical Activity

The 24-hour recall method was used to estimate the level of physical activity and reported in metabolic equivalents × hours per day (Met.h/d). Activity levels were categorized into four classes (light, moderate, vigorous, and intense). The level of physical activity of subjects was presented as Met.h/d [28].

### 2.9. Statistical Analysis

The general characteristics of the population were compared in tertiles of the PD and MD using *χ*^2^ tests and one-way ANOVA of variance for categorical and continuous variables, respectively. The dietary intakes of participants were computed using the ANCOVA analysis and by adjusting for the energy intake among tertiles of the PD and MD.

Psychological profile variables were analyzed as both continuous and categorical variables. The mean scores of depressions, anxiety, and stress were compared in tertiles of the PD and MD using the ANCOVA test. Furthermore, we used the binary logistic regression to provide odds ratios (OR) and 95% confidence intervals (95% CI), in crude and adjusted models, for the association of the PD and MD with psychological profile variables, in which the median was considered as a cut-point to categorize variables into two groups. According to previous publications, factors such as energy intake, age, physical activity, socioeconomic status, marital, and dietary supplement use were considered confounding variables. SPSS version 24 (SPSS Inc, Chicago, IL, USA) was applied to perform statistical analysis. *P* < 0.05 was considered statistically significant. The PD and MD variables are both independent variables.

## 3. Results

Participants' baseline characteristics by diet score tertiles are demonstrated in Table 2. Individuals' mean age and the BMI were 31.37 years and 23.78 kg/m^2^, respectively. There was no significant difference between the tertiles of the PD and MD and most of the variables. Only a significant association was observed between the PD and participants' socioeconomic status.

The energy-adjusted intake of selected nutrients and food groups across tertiles of the PD and MD are shown in Tables 3 and 4. Compared with those in the first tertile, individuals in the third tertile of the PD had a higher intake of energy, protein, carbohydrate, fiber, vitamin B6, folic acid, calcium, magnesium, zinc, fruits, vegetables, nuts, and fish and lower intakes of fat, sodium, grain and starch, red and processed meat, and sugar-sweetened beverages. Furthermore, higher adherence to the MD score was associated with higher intakes of energy, fiber, vitamin B6, folic acid, magnesium, fruits, vegetables, nuts, and fish and lower intakes of sodium, grain, starch, and red and processed meat.

Multivariable-adjusted means and standard errors (SE) of depression, anxiety, and stress scores for each tertile of both dietary pattern scores are provided in Table 5. In the crude model and after adjusting for potential confounders such as age, BMI, energy intake, physical activity, socioeconomic status, marital status, educational status, and supplement use, women in the highest tertile of the PD had lower depression, anxiety, psychological stress score than those in the lowest tertile. This association also was observed between psychological profile variables and MD.

In the crude model, participants in the top tertile of the PD had a lower risk of depression (OR = 0.26; 95% CI: 0.16, 0.43: *P* ≤ 0.001), anxiety (OR = 0.32; 95% CI: 0.20, 0.51: *P* ≤ 0.001), and stress (OR = 0.23; 95% CI: 0.14, 0.38: *P* ≤ 0.001). After controlling for potential confounders, this significant association was even strengthened (depression: OR = 0.21; 95% CI: 0.12, 0.37: *P* ≤ 0.001; anxiety: OR = 0.27; 95% CI: 0.16, 0.45: *P* ≤ 0.001; and stress: OR = 0.19; 95% CI: 0.11, 0.32; *P* ≤ 0.001) (Table 6). In crude and fully adjusted models, women with the highest tertile of the MD score had a lower odds of depression (Crude model: OR = 0.25; 95% CI: 0.15, 0.42; *P* ≤ 0.001 and model 2: OR = 0.20; 95% CI: 0.11, 0.36; *P* ≤ 0.001), anxiety (Crude model: OR = 0.28; 95% CI: 0.17, 0.45; *P* ≤ 0.001 and model 2: OR = 0.22; 95% CI: 0.13, 0.38; *P* ≤ 0.001), and stress (Crude model: OR = 0.29; 95% CI: 0.18, 0.48; *P* ≤ 0.001 and model 2: OR = 0.23; 95% CI: 0.13, 0.39; *P* ≤ 0.001) compared with those in the lowest tertile (Table 6).

## 4. Discussion

Our finding supposed that women following the PD and MD dietary patterns had a lower risk of psychological disorders such as depression, anxiety, and stress. To the best of our knowledge, the current study is the first investigation that evaluates the association of the Paleolithic diet with psychological profiles among a sample of an Iranian women.

Several putative mechanisms may explain the protective effects of Paleolithic and Mediterranean diet patterns on psychological profiles. One of the factors that have a relationship to psychological disorders is inflammation and oxidative stress [29, 30]. Some food groups high in Paleolithic and Mediterranean diets, such as vegetables, fruits, and nuts, are rich sources of antioxidants [31]. Thus, they could improve inflammation and oxidative balance.

Moreover, these food groups are high in fiber and micronutrients such as magnesium, folic acid, and vitamin C. The intake of fiber, particularly from vegetables and fruits, has an inverse association with symptoms of depression [32]. Dietary fibers such as pectin, gums, and fructans cannot be digested by human enzymes and play a role as a prebiotic. They may be fermented by microbial flora and produce short-chain fatty acids (SCFAs) [33]. SCFAs are able to improve intestinal epithelial barrier integrity, consequently decreasing permeability to lipopolysaccharides that are an endotoxin, release from pathogen bacteria, and increase inflammation [34]. Moreover, prebiotic fibers can help a healthier microbiome in the gastrointestinal tract [35]. The intestinal microbiome has an important effect on the gut-brain axis that plays a crucial role in mental health and brain function [36].

Magnesium is an essential element that not only plays an important role in numerous reactions in the neuron system as a coenzyme but also has an antidepressant effect through N-methyl-D-aspartate (NMDA) receptor block [37]. Folic acid is also associated with brain function via the effect on the metabolism of biogenic amines such as serotonin [38]. Vitamin C could improve mental function through several mechanisms, such synthesizing neurotransmitters, neuronal maturation, and anti-inflammatory activities [39].

The Paleolithic and Mediterranean diet patterns emphasize lower consumption of saturated fatty acids and higher consumption of monounsaturated fatty acids and polyunsaturated fatty acids, especially *n*-3 PUFAs. The central nervous system has the most percentage of lipids in the body after adipose tissue [40]. The dietary fatty acid composition can affect brain function [41]. The intake of saturated fatty acids (SFAs) could lead to neuroinflammation and impair brain activity [42, 43]. On the other hand, a higher intake of monounsaturated fatty acids (MUFAs) may promote insulin signaling in the brain and preserve the integrity of the brain's dopamine system, consequently diminishing the risk of depression [43, 44]. Moreover, *n*-3 PUFAs could affect the function of the brain by regulating brain-derived neurotropic factor BDNF, synthesis of the neurotransmitter, and synaptic plasticity [45].

Our findings revealed that adhering to the Paleolithic and Mediterranean diets is related to a lower risk of depression, anxiety, and stress. There are few studies that assess the relationship of the Paleolithic diet to psychological variables, and only a study by Norwood and collaborators evaluated the effect of the Paleolithic diet on psychological characteristics in 42 people (94% females) [46]. The Paleolithic diet was considered a restrictive dietary pattern in the mentioned study. They concluded that individuals following a Paleolithic diet had better psychological well-being compared to people who did not follow a particular diet. As mentioned, oxidative stress and inflammation may be essential in psychological disorders. A cross-sectional study of 646 men and women aged 30 to 74 years old showed that following Paleolithic and Mediterranean diet patterns is inversely associated with lower oxidative stress and inflammation [47]. However, several prior studies examined the association of the Mediterranean diet with psychological variables, but there are limited investigations that have been conducted in the Middle East area, particularly in women. A cross-sectional study by Sadeghi et al. showed an inverse association between adherence to the Mediterranean diet with depression, anxiety, and stress [48]. Another study evaluated the association of the Mediterranean diet with psychological disorders in female adolescents [16]. Like our study, the score of psychological disorders, including depression, anxiety, and stress, were determined by DASS-21 questionnaire. Their results showed that a higher score of the Mediterranean diet was associated with a reduced risk of depression but no significant association with anxiety and stress. Furthermore, a recent updated meta-analysis of observational studies by Shafiei et al. found that there is no significant association between the following Mediterranean diet and risk of depression based on the analysis of cohort studies; however, a significant inverse association was observed between adherence to the Mediterranean diet and depression risk in cross-sectional studies [27].

Our study has several strengths, including adjusting for several potential confounders, interviewing participants by expert nutritionists, and using a valid and reliable FFQ for collecting nutritional data. However, it is necessary to consider some limitations. It is impossible to characterize a causality relationship given the study's cross-sectional design. The study population was adult women 20–50 years old, and it may not be correct to generalize our findings to other populations with different conditions. In addition, for the assessment of the psychological profile, the DASS-21 questionnaire was used. The DASS-21 questionnaire is suitable for screening depression, anxiety, and stress and could not be valid for clinical diagnosis.

Our finding supposes that following Paleolithic or Mediterranean dietary patterns could be inversely associated with psychological disorders such as depression, anxiety, and stress in a sample of adult women. Further studies with large-scale and prospective cohorts or intervention designs are needed to deepen our understanding of the effect of Paleolithic or Mediterranean dietary patterns on mental health.

## Figures and Tables

**Table 2:** Mediterranean diet Paleolithic diet score measurement.

Intake category	Scoring	Mediterranean diet score	Paleolithic diet score
Maximum intake “best”	Number of points assigned to each quintile equals quintile rank (for example, the highest and lowest quintiles received +5 and +1 points, respectively)	Vegetable, fruit, lean meat, fish, and nut Monounsaturated: saturated fat ratio	Vegetable, fruit, fruit and vegetable diversity, lean meat, fish, nut

Lowest intake	Reverse quintile rank = number of points allocated to each quintile (e.g., the top and lowest quintiles received +1 and +5 points, respectively)	Red meat and processed meat, sodium (mg)	Calcium, red meat and processed meat, sodium, dairy food grain and starches, baked good sugar-sweetened beverages

Moderate intake	The third quintile accumulated +5 points, the second and fourth quintiles accumulated +3 points, and the first and fifth quintiles accumulated +1 point	Dairy foods grain and starches	

**Table 1:** General characteristics of participants across the tertiles of PD and MD scores.

Variable	Total 435	PD			*P* value	MD			*P* value
		*T*1 152	*T*2 132	*T*3 151		*T*1 132	*T*2 147	*T*3 156	
Age (year)	31.37 ± 7.52	30.67 ± 7.64	31.26 ± 7.53	32.18 ± 7.35	0.214	30.77 ± 7.31	31.70 ± 7.69	31.58 ± 7.54	0.536
BMI (kg/m^2^)	23.78 ± 4.10	23.82 ± 4.19	23.28 ± 3.83	24.16 ± 4.21	0.191	23.69 ± 3.87	23.83 ± 4.44	23.80 ± 3.97	0.985
Physical activity (Met.h/d)	39.79 ± 6.58	38.94 ± 6.95	40.06 ± 6.22	40.39 ± 6.47	0.135	39.13 ± 6.84	39.79 ± 6.69	40.34 ± 6.24	0.299
SES (*n* (%))					0.032				0.619
Low	165 (37.9%)	70 (42.4%)	47 (28.5%)	48 (29.1%)		56 (33.9%)	57 (34.5%)	52 (31.5%)	
Medium	109 (25.0%)	39 (35.8%)	28 (25.7%)	42 (38.5%)		31 (28.4%)	37 (33.9%)	41 (37.6%)	
High	161 (37.1%)	43 (26.7%)	57 (35.4%)	61 (37.9%)		45 (28.0%)	53 (32.9%)	63 (39.1%)	
Marital status (*n* (%))					0.415				0.709
Single	183 (42.0%)	64 (35.0%)	61 (33.3%)	58 (31.7%)		52 (28.4%)	62 (33.9%)	69 (37.7%)	
Married	252 (58.0%)	88 (34.9%)	71 (28.2%)	93 (36.9%)		80 (31.7%)	85 (33.7%)	87 (34.5%)	
Educational status (*n* (%))					0.770				0.338
≤Diploma	139 (31.9%)	51 (36.7%)	43 (30.9%)	45 (32.4%)		45 (32.4%)	51 (36.7%)	43 (30.9%)	
>Diploma	296 (68.1%)	101 (34.1%)	89 (30.1%)	106 (35.8%)		87 (29.4%)	96 (32.4%)	113 (38.2%)	
Supplement use (*n* (%))					0.106				0.955
Yes	163 (37.5%)	50 (30.7%)	59 (36.2%)	54 (33.1%)		50 (30.7%)	56 (34.4%)	57 (35.0%)	
No	272 (62.5%)	102 (37.5%)	73 (26.8%)	97 (35.7%)		82 (30.1%)	91 (33.5%)	99 (36.4%)	

PD, Paleolithic diet; MD, Mediterranean diet; SES, socioeconomic status. Values are mean (SD) for continuous variables and percentage for dichotomous variables. Using one-way ANOVA for continuous variables and chi-square test for categorical variables.

**Table 3:** Dietary intakes of study participants across the tertiles of MD scores.

Variable	MD			*P* value
	*T*1 132	*T*2 147	*T*3 156	
Energy (kcal/d)	1861.11 ± 42.84	2123.02 ± 40.60	2211.85 ± 39.41	<0.001

*Nutrients*				
Protein (g/d)	74.69 ± 1.33	73.35 ± 1.22	76.24 ± 1.20	0.243
Fat (g/d)	77.17 ± 1.19	74.13 ± 1.10	76.41 ± 1.08	0.142
Carbohydrate (g/d)	285.49 ± 2.77	292.63 ± 2.56	288.70 ± 2.51	0.167
Fiber (g/d)	14.74 ± 0.41	16.03 ± 0.38	18.17 ± 0.37	<0.001
Vitamin B6 (mg/d)	1.19 ± 0.02	1.27 ± 0.02	1.45 ± 0.02	<0.001
Folic acid (*µ*g/d)	286.34 ± 7.00	306.11 ± 6.46	336.25 ± 6.34	<0.001
Calcium (mg/d)	1057.13 ± 24.79	1007.04 ± 22.88	1028.24 ± 22.48	0.338
Sodium (mg/d)	6108.79 ± 255.83	5332.74 ± 236.07	4552.06 ± 231.95	<0.001
Magnesium (mg/d)	248.77 ± 4.27	256.44 ± 3.94	280.03 ± 3.87	<0.001
Zinc (mg/d)	8.77 ± 0.26	8.57 ± 0.24	9.32 ± 0.24	0.082
Fe (mg/d)	21.79 ± 1.87	19.83 ± 1.73	23.23 ± 1.70	0.370

*Food groups*				
Grain and starch (g/d)	360.48 ± 9.17	362.80 ± 8.46	320.43 ± 8.31	<0.001
Fruits (g/d)	224.05 ± 15.85	267.35 ± 14.63	326.37 ± 14.37	<0.001
Vegetable (g/d)	246.17 ± 14.75	307.39 ± 13.61	375.90 ± 13.37	<0.001
Nuts (g/d)	8.90 ± 1.08	10.51 ± 1.00	15.33 ± 0.98	<0.001
Red and processed meat (g/d)	43.75 ± 2.61	31.84 ± 2.41	26.94 ± 2.37	<0.001
Fish (g/d)	6.27 ± 1.51	7.95 ± 1.39	13.89 ± 1.37	<0.001
Dairy (g/d)	480.75 ± 18.73	432.85 ± 17.29	444.69 ± 16.98	0.161
Sugar-sweetened beverages (g/d)	20.18 ± 4.00	20.41 ± 3.69	9.69 ± 3.62	0.068

MD, Mediterranean diet. Values are mean ± SE. All values are adjusted for energy intake using ANCOVA.

**Table 4:** Dietary intakes of study participants across the tertiles of PD scores.

Variable	PD			*P* value
	*T*1 152	*T*2 132	*T*3 151	
Energy (kcal/d)	1951.86 ± 40.99	2122.32 ± 43.98	2158.75 ± 41.12	0.001

*Nutrients*				
Protein (g/d)	73.36 ± 1.20	72.57 ± 1.28	78.18 ± 1.20	0.002
Fat (g/d)	78.11 ± 1.08	76.21 ± 1.15	73.32 ± 1.08	0.008
Carbohydrate (g/d)	282.20 ± 2.51	290.51 ± 2.67	294.67 ± 2.51	0.002
Fiber (g/d)	13.69 ± 0.34	16.10 ± 0.36	19.41 ± 0.34	<0.001
Vitamin B6 (mg/d)	1.18 ± 0.02	1.28 ± 0.02	1.47 ± 0.02	<0.001
Folic acid (*µ*g/d)	273.59 ± 6.00	304.05 ± 6.38	354.50 ± 5.99	<0.001
Calcium (mg/d)	983.66 ± 22.50	1021.27 ± 23.92	1083.83 ± 22.44	0.007
Sodium (mg/d)	5820.35 ± 236.33	5368.66 ± 251.31	4682.37 ± 235.77	0.003
Magnesium (mg/d)	239.53 ± 3.64	256.67 ± 3.87	290.93 ± 3.63	<0.001
Zinc (mg/d)	8.52 ± 0.24	8.55 ± 0.25	9.60 ± 0.24	0.002
Fe (mg/d)	19.33 ± 1.71	21.03 ± 1.82	24.51 ± 1.70	0.096

*Food groups*				
Grain and starch (g/d)	374.68 ± 8.35	345.34 ± 8.88	320.30 ± 8.33	<0.001
Fruits (g/d)	192.94 ± 13.88	283.18 ± 14.76	351.54 ± 13.85	<0.001
Vegetable (g/d)	228.92 ± 12.37	283.31 ± 13.15	424.70 ± 12.34	<0.001
Nuts (g/d)	9.29 ± 1.00	12.08 ± 1.07	13.94 ± 1.00	0.005
Red and processed meat (g/d)	41.74 ± 2.40	31.27 ± 2.55	27.73 ± 2.40	<0.001
Fish (g/d)	7.01 ± 1.39	8.23 ± 1.47	13.31 ± 1.38	0.004
Dairy (g/d)	438.58 ± 17.20	457.20 ± 18.30	459.89 ± 17.16	0.643
Sugar-sweetened beverages (g/d)	29.04 ± 3.59	15.71 ± 3.82	4.55 ± 3.58	<0.001

PD, Paleolithic diet. Values are mean ± SE. All values are adjusted for energy intake using ANCOVA.

**Table 5:** Mean of mental health scores across tertiles of PD and MD scores.

Variable	PD			*P* value^d^	MD			*P* value
	*T*1 152	*T*2 132	*T*3 151		*T*1 132	*T*2 147	*T*3 156	
*Depression*								
Crude	13.01 ± 0.80^c^	9.54 ± 0.86	5.41 ± 0.81	<0.001	13.27 ± 0.87	8.83 ± 0.83	6.44 ± 0.80	<0.001
Model 1^a^	13.42 ± 0.79	9.57 ± 0.84	4.97 ± 0.79	<0.001	13.99 ± 0.87	8.61 ± 0.80	6.04 ± 0.79	<0.001
Model 2^b^	13.30 ± 0.79	9.42 ± 0.84	5.20 ± 0.79	<0.001	13.88 ± 0.87	8.52 ± 0.80	6.21 ± 0.79	<0.001

*Anxiety*								
Crude	11.17 ± 0.70	9.01 ± 0.76	5.40 ± 0.71	<0.001	11.13 ± 0.76	8.72 ± 0.72	6.10 ± 0.70	<0.001
Model 1	11.47 ± 0.70	9.07 ± 0.74	5.04 ± 0.69	<0.001	11.71 ± 0.77	8.56 ± 0.71	5.76 ± 0.70	<0.001
Model 2	11.41 ± 0.71	9.03 ± 0.75	5.09 ± 0.71	<0.001	11.65 ± 0.77	8.49 ± 0.71	5.85 ± 0.70	<0.001

*Stress*								
Crude	18.26 ± 0.75	14.40 ± 0.81	10.50 ± 0.76	<0.001	18.15 ± 0.82	14.09 ± 0.78	11.51 ± 0.76	<0.001
Model 1	18.52 ± 0.75	14.51 ± 0.79	10.14 ± 0.74	<0.001	18.70 ± 0.83	13.94 ± 0.76	11.18 ± 0.75	<0.001
Model 2	18.49 ± 0.75	14.38 ± 0.80	10.22 ± 0.75	<0.001	18.60 ± 0.83	13.81 ± 0.77	11.35 ± 0.75	<0.001

PD, Paleolithic diet; MD, Mediterranean diet. ^a^ Adjusted for BMI, age, and energy intake. ^b^ Additionally adjusted for socioeconomic status, physical activity, marriage status, educational status, and supplement use. ^c^ These values are mean ± SE. ^d^ Using ANCOVA.

**Table 6:** Crude and multivariable-adjusted odds ratios and 95% CIs for psychological disorders across tertiles of PD and MD scores.

Variable	PD			*P* trend^d^	MD			*P* trend
	*T*1 152	*T*2 132	*T*3 151		*T*1 132	*T*2 147	*T*3 156	
*Depression*								
Crude	1.00	0.54 (0.33, 0.87)^c^	0.26 (0.16, 0.43)	<0.001	1.00	0.43 (0.26, 0.69)	0.25 (0.15, 0.42)	<0.001
Model 1^a^	1.00	0.50 (0.30, 0.83)	0.21 (0.12, 0.37)	<0.001	1.00	0.35 (0.21, 0.59)	0.20(0.11, 0.35)	<0.001
Model 2^b^	1.00	0.48 (0.29, 0.81)	0.21 (0.12, 0.37)	<0.001	1.00	0.34 (0.20, 0.57)	0.20 (0.11, 0.36)	<0.001

*Anxiety*								
Crude	1.00	0.63 (0.39, 1.01)	0.32 (0.20, 0.51)	<0.001	1.00	0.55 (0.34, 0.88)	0.28 (0.17, 0.45)	<0.001
Model 1	1.00	0.60 (0.37, 0.99)	0.27 (0.16, 0.44)	<0.001	1.00	0.46 (0.27, 0.76)	0.22 (0.12, 0.37)	<0.001
Model 2	1.00	0.62 (0.37, 1.02)	0.27 (0.16, 0.45)	<0.001	1.00	0.45 (0.27, 0.75)	0.22 (0.13, 0.38)	<0.001

*Stress*								
Crude	1.00	0.49 (0.31, 0.79)	0.23 (0.14, 0.38)	<0.001	1.00	0.44 (0.27, 0.72)	0.29 (0.18, 0.48)	<0.001
Model 1	1.00	0.46 (0.28, 0.75)	0.19 (0.11, 0.33)	<0.001	1.00	0.37 (0.22, 0.61)	0.23 (0.13, 0.39)	<0.001
Model 2	1.00	0.42 (0.25, 0.70)	0.19 (0.11, 0.32)	<0.001	1.00	0.35 (0.21, 0.59)	0.23 (0.13, 0.39)	<0.001

PD, Paleolithic diet; MD, Mediterranean diet. ^a^ Adjusted for BMI, age, and energy intake. ^b^ Additionally adjusted for socioeconomic status, physical activity, marriage status, educational status, and supplement use. ^c^ These values are odds ratios (95% CIs). ^d^ Obtained from logistic regression.

## Data Availability

Data are available from the corresponding author upon reasonable request.
